# Indications and selection of MR enterography vs. MR enteroclysis with emphasis on patients who need small bowel MRI and general anaesthesia: results of a survey

**DOI:** 10.1007/s13244-015-0384-2

**Published:** 2015-04-09

**Authors:** Michael R. Torkzad, Gabriele Masselli, Steve Halligan, Aytek Oto, Henning Neubauer, Stuart Taylor, Arun Gupta, Jens Brøndum Frøkjær, Ian C. Lawrance, Christopher J. Welman, Anne Negård, Olle Ekberg, Michael Patak, Thomas Lauenstein

**Affiliations:** 1 Department of Radiology, Oncology, Radiation Sciences, Uppsala University, Uppsala, Sweden; 2 Umberto I Hospital, Radiology Department, Sapienza University, Rome, Italy; 3 Center for Medical Imaging, University College London, London, UK; 4 Department of Radiology, University of Chicago, Chicago, IL USA; 5 Department of Paediatric Radiology, University Hospital Wuerzburg, Wuerzburg, Germany; 6 Center for Medical Imaging, University College London, London, UK; 7 Intestinal Imaging, St Mark’s Hospital, Harrow, UK; 8 Department of Radiology, Aalborg University Hospital, Aalborg, Denmark; 9 School of Medicine and Pharmacology, University of Western Australia and Centre for Inflammatory Bowel Disease, Fremantle Hospital, Fremantle, Australia; 10 Department of Radiology, Fremantle Hospital, Fremantle, Australia; 11 Abdominal and Children’s Radiology, Department of Radiology, Akershus University Hospital, Lørenskog, Norway; 12 Department of Clinical Sciences/Medical Radiology, Lund University; Diagnostic Centre of Imaging and Functional Medicine Lund University Skåne University Hospital, Malmö, Sweden; 13 Klinik Hirslanden, Witellikerstrasse 40, Zürich, Austria; 14 Department of Diagnostic and Interventional Radiology and Neuroradiology, University Hospital Essen, Essen, Germany

**Keywords:** Small bowel, MRI, Crohn’s disease, General anaesthesia

## Abstract

**Aims:**

To survey the perceived indications for magnetic resonance imaging of the small bowel (MRE) by experts, when MR enteroclysis (MREc) or MR enterography (MREg) may be chosen, and to determine how the approach to MRE is modified when general anaesthesia (GA) is required.

**Materials and methods:**

Selected opinion leaders in MRE completed a questionnaire that included clinical indications (MREg or MREc), specifics regarding administration of enteral contrast, and how the technique is altered to accommodate GA.

**Results:**

Fourteen responded. Only the diagnosis and follow-up of Crohn’s disease were considered by over 80 % as a valid MRE indication. The remaining indications ranged between 35.7 % for diagnosis of caeliac disease and unknown sources of gastrointestinal bleeding to 78.6 % for motility disorders. The majority chose MREg over MREc for all indications (from 100 % for follow-up of caeliac disease to 57.7 % for tumour diagnosis). Fifty per cent of responders had needed to consider MRE under GA. The most commonly recommended procedural change was MRI without enteral distention. Three had experience with intubation under GA (MREc modification).

**Conclusion:**

Views were variable. Requests for MRE under GA are not uncommon. Presently most opinion leaders suggest standard abdominal MRI when GA is required.

***Main messages*:**

• *Experts are using MRE for various indications*.

• *Requests for MRE under general anaesthesia are not uncommon*.

• *Some radiologists employ MREc under general anaesthesia*; *others do not distend the small bowel*.

## Introduction

In a recent evidence-based consensus for assessment of small bowel (SB) inflammatory disease [1] jointly from the European Crohn’s and Colitis Organisation (ECCO) and European Society of Gastrointestinal and Abdominal Radiology (ESGAR), it was stated that (abbreviation added to suit our article): “Magnetic resonance imaging (MRI) of the small bowel (MRE) and colon requires fast imaging techniques and luminal distension [Evidence Level (EL) 2]. MRE has similar diagnostic accuracy and similar indications to CT, but with the major advantage of not imparting ionising radiation [EL 1]”.

While most radiologists recommend distention for adequate SB assessment, methods to achieve this vary. Some use enterography (oral administration) or enteroclysis (nasojejunal intubation with the tube tip in close proximity to the ligament of Treitz). Improved capability of MRI to diagnose SB pathology has generated increasing requests to perform these examinations in patients who require general anaesthesia (GA). Demand is particularly increasing in the paediatric age group, not least in an attempt to limit exposure to ionising radiation. SB distension with large volumes of orally administered fluids, however, is generally considered a contraindication in GA, or heavy sedation, given the risk of aspiration [2–5]. To deliberately distend the SB with fluids would, therefore, be contradictory to this general rule.

To date there are no published data on the safety or otherwise of performing MRE under GA, nor is there any guidance on any required protocol modifications.

The purposes of this study were to:

1. Survey key opinion leaders regarding their current indications and protocols for undertaking MRE.
2. To document their attitudes and experience of performing MRE under GA in order to issue guidance for this specific clinical scenario.

## Materials and methods

Two radiologists (MRT and TL) devised the questionnaire, which covered four broad topic areas addressed by a series of multiple choice questions, together with space for free comments.

The questionnaire was sent to recognised opinion leaders, chosen via a history of prior indexed publications, supplemented by the chief investigators personal knowledge of their work and contributions in the field. Twenty-four radiologists were identified initially and each contacted on up to three occasions for an initial response with a goal to recruit at least ten completed responses. If responses were incomplete, participants were again contacted. The topic areas addressed were as follows:

- Part I (questions 1–12): The first topic covered the number of examinations performed annually and the imaging modality of choice for named different pathologies with an emphasis on magnetic resonance enterography (MREg) vs. magnetic resonance enteroclysis (MREc).
- Part II (questions 13–20): The preference(s) of opinion leaders for SB distension including type of contrast agent, desired signal characteristics, and mode and rate of administration.
- Part III (questions 21–25): Experience with MRI under GA was canvassed including any changes in indications in comparison to routine MRE. Specifically experts were asked if they favored MRI without SB distension, instead of MRE when GA was required, and whether they would rather choose an alternative modality (e.g. CT).
- Part IV (questions 26–39): These related to protocol modifications made by experts if they performed SB MRI under GA.

The results were entered in Excel file sheets and expressed as percentages of the responses.

## Results

Fourteen experts completed the questionnaire. Two of the invitees were not radiologists and chose not to participate, and two others mentioned that they were working at the same institution as the responding 14 and wanted to avoid duplication. The other six invitees did not respond. Among the responders, all interpreted between 100 and 500 small bowel MRIs annually, averaging 222.

### Part I

**Fig. 1:**
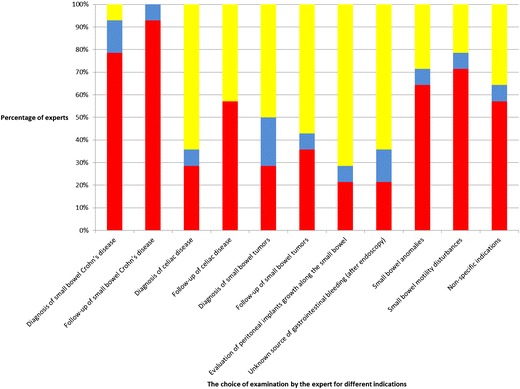
The choice of examinations by different experts for different diseases. *Blue bars*: MREc or MR enterlocysis; *red bar*: MREg or MR enterography; *yellow bar*: other free choices of both radiological imaging methods or any other possible means

All experts (14 out of 14 or 100 %) advocate MRI for follow-up of SB Crohn’s disease with most advocating MREg (13 out of 14, 93 %) as opposed to MREc (1 out of 14, Fig. 1). The majority used MRI to confirm/refute an initial diagnosis of SB Crohn’s disease with 93 % (13 out of 14) choosing MRE (of whom 11 or 79 % chose MREg).

For studying SB motility disorders 79 % (11 out of 14) chose MRE (10 out of 14 or 71 % MREg).

The most frequent indications where MRE (MREg or MREc) was ***not chosen*** by the majority of experts as the imaging modality of choice were (followed by the proportion who preferred other radiologic and non-radiologic methods of valuation): the evaluation of serosal tumour implants (10 out of 14 or 71 %), diagnosis of caeliac disease (9 out of 14 or 64 %), unknown source of gastrointestinal bleeding after negative colonoscopy and gastroduodenoscopy (9 out of 14 or 64 %), and follow-up (8 out of 14 or 57 %) and diagnosis (7 out of 14 or 50 %) of SB tumours (Fig. 1). In all the other conditions, as mentioned in Fig. 1, MRE was preferred.

Interestingly, MREg was always more frequently favoured than MREc, between 0 % (follow-up of caeliac disease with MREc and MREg favoured by none and 8 experts, respectively) to 43 % (diagnosis of small bowel tumours with MREc and MREg favoured by 3 and 4 experts, respectively).

### Part II

#### Position of the patient

The prone position was clearly favoured (12 experts out of 14 or 86 %), with supine positioning as second (2 experts out of 14 or 14 %).

#### Type of oral contrast agent

Among the contrast agents specified, mannitol was the most common among the experts (4 experts out of 14 or 29 %), followed by VoLumen (3 experts out of 14 or 21 %), sorbitol (2 experts out of 14 or 14 %) and tap water by one expert (1 out of 14 or 7 %). Four experts chose other contrast agents [locust bean gum (LBG) by two researchers and polyethylene glycol solution (PEG) and Glycoprep-C at one centre each]. The most common feature among all experts was their choice of contrast agents that had the signal characteristics of water, i.e. double phasic being high signal on T2 weighting and low signal on T1 weighting.

#### Factors influencing the rate and volume of the administered contrast agent

**Fig. 2:**
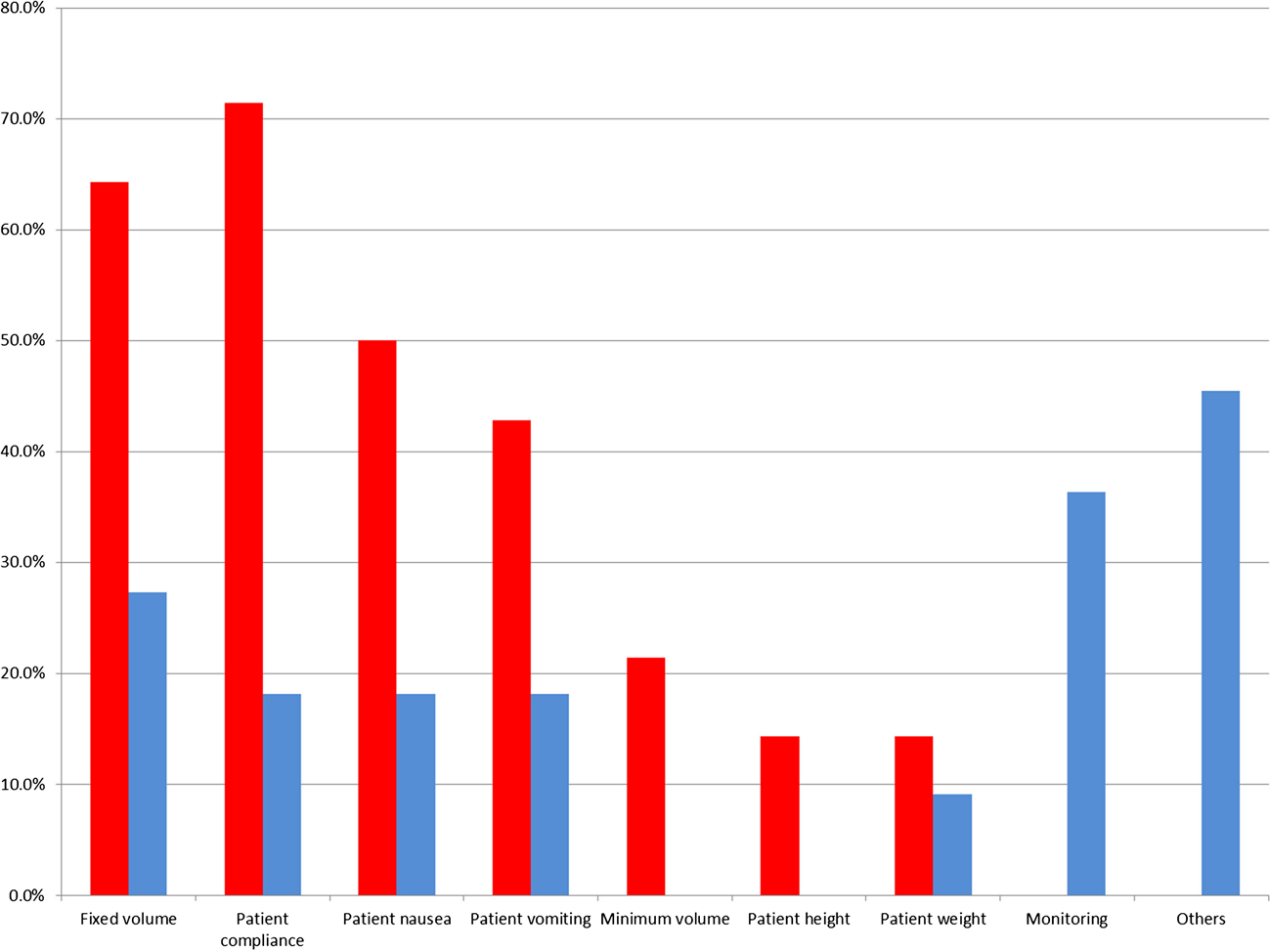
Factors influencing the volume of enteral contrast agents. *Blue bars*: MREc or MR enterlocysis; *red bar*: MREg or MR enterography

**Fig. 3:**
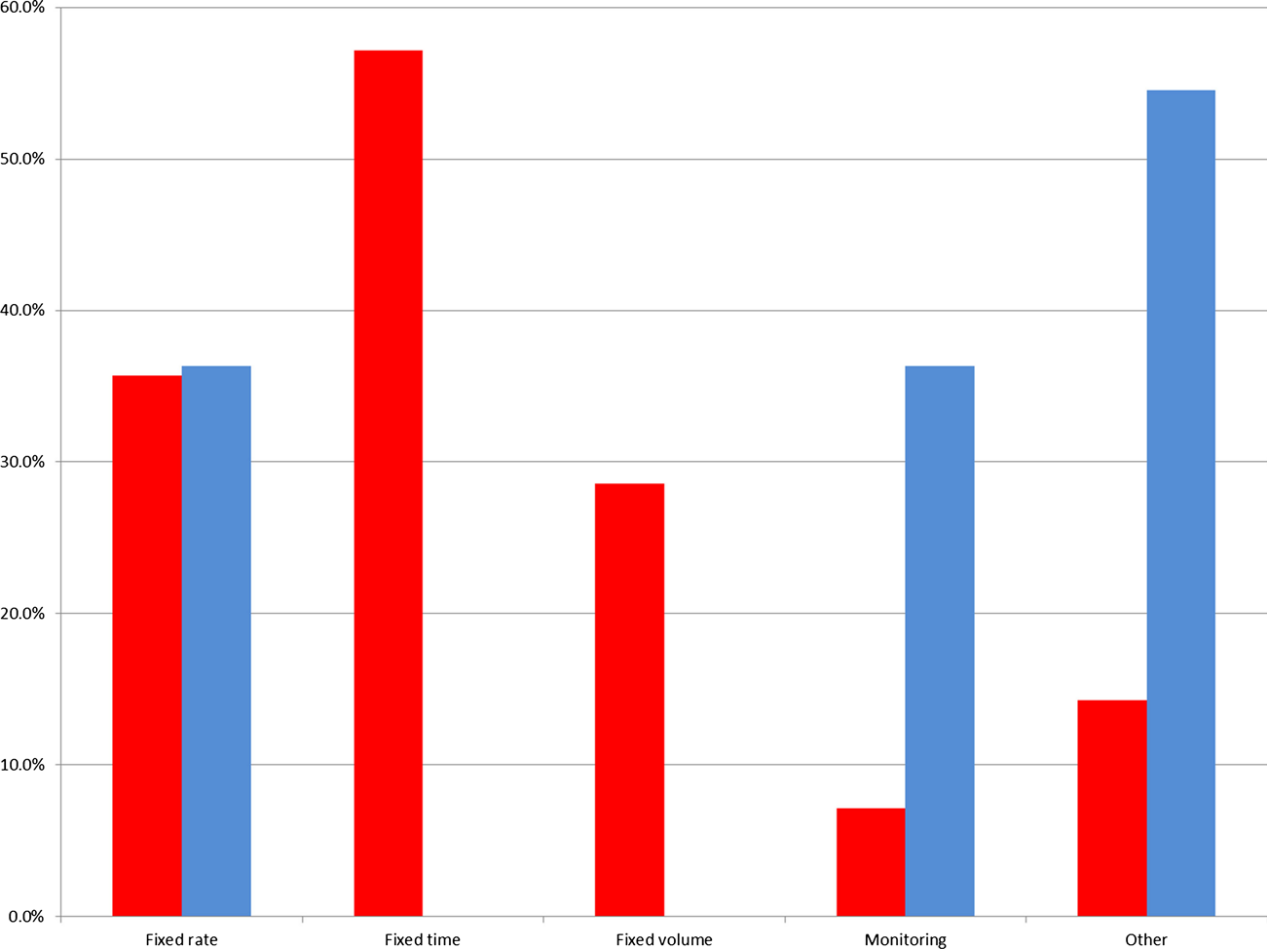
Factors influencing the rate of administration of enteral agent. *Blue bars*: MREc or MR enterlocysis; *red bar*: MREg or MR enterography

The volume of oral agent ingested during MREg was influenced primarily by patient compliance, even though a fixed volume was planned to be given at the start of the procedure. The time allowed for ingestion was always fixed and thus it was the rate of administration that resulted in the differences in administered oral agent volumes. For MREc, monitoring progression of the contrast agent with a fixed rate seemed to be most influential in the determination of the administration rate (Figs. 2 and 3).

### Part III

MRI under GA was performed only at 8 out of 14 (57 %) of the centres. In half of the centres with (4 out of 14) and half of those without (3 out of 14), the possibility of MRI under GA had led to the request to perform MRE under GA.

**Fig. 4:**
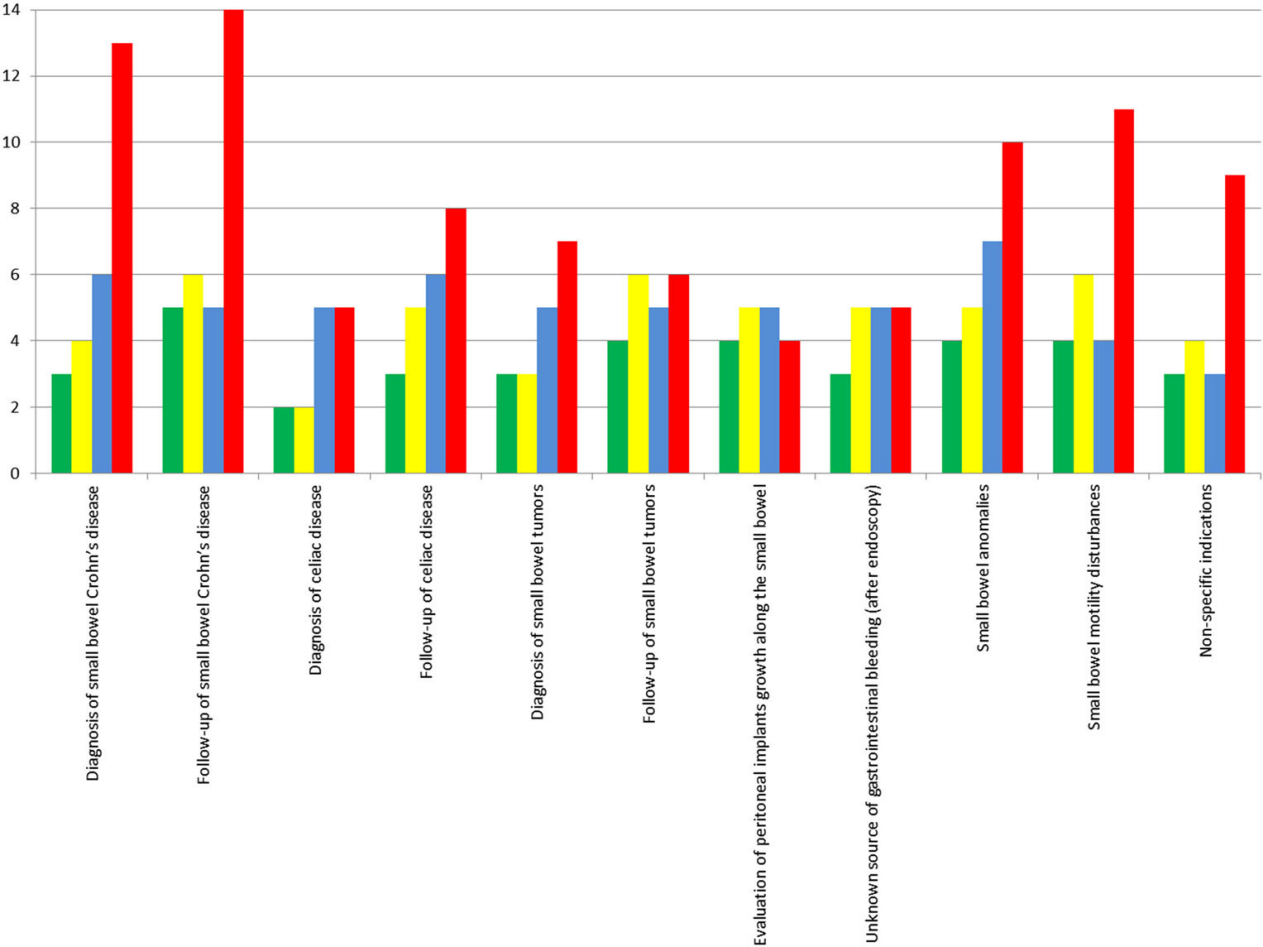
Change in methodology in the number of experts when confronted with the dilemma of doing MRI under general anaesthesia. *Green bar*: MRE is not the best alternative (among those with access to MRI under general anaesthesia). *Yellow bar*: MRE is not the best alternative (among those with or without access to MRI under GA). *Blue bar*: distention is not essential and therefore MRI of the abdomen is sufficient. *Red bar*: how participant answered to indications for MRE in part I

When asked to do MRE under GA 3 out of 14 (21 %) experts did not recommend MRI (Fig. 4). Eight experts out of 14 (57 %), however, would perform standard abdominal MRI (4 out of 14 or 29 %), e.g. with no SB distension or choosing some form of intubation (4 out of 14 or 29 %). Intubation would be done either as an MREc alone or an MREc with the addition of a stomach tube to drain excessive stomach fluid. Three experts perform MRE under GA.

### Part IV

This section dealt with modifications employed if there was a question of combining MRE with a GA. Most cases where GA is requested are young children who cannot comply with the examination. However there are some patients who require GA because of impaired cognitive function or mental retardation. Patient age has an important role. The number of experts employing prone positioning decreased to 6 out of 14 (41 %), 3 choosing supine, 3 choosing whatever position was easiest for the anaesthesiologist and one choosing right decubitus. It should be considered that a supine patient is in the most convenient position for the anaesthesiologist unless specific issues warrant other positions.

An almost equal number of experts, 4 out of 14 (27 %) and 3 out of 14 (36 %), employed endoscopic methods and/or fluoroscopy for nasojejunal tube insertion if enteroclysis would be employed under these circumstances, respectively.

Of the experts questioned only three had first-hand experience with MRE under GA (initials blinded). Most of the differences in their protocols are related to differences in MREc in general. The major differences among the authors that are related to MREc being done under GA is the use of one tube (*n* = 2 participants) or two tubes (*n* = 1 participant) method. The latter participant tries to combine upper gastrointestinal endoscopy under GA (which patients usually need for various reasons) with MREc under GA. The endoscopist inserts both tubes with the tip in the stomach, and the radiologist then advances one tube to its position at the level of the Treitz ligament. He or she uses two nasogastric tubes for drainage of any regurgitated fluids from the intestines into the stomach during MRE.

## Discussion

This survey illustrates a degree of discrepancy among experts performing MRE. Considering the proportion agreeing on a valid indication for MRE, only two indications exceeded 80 %: the initial diagnosis and follow-up of Crohn’s disease [6–18]. Otherwise, we observed a wide variation among our experts regarding other indications for MRE. These ranged from 29 % suggesting MRE for evaluation of serosal carcinomatosis (oral presentation at the European Congress of Radiology 2014 by MRT) and ischaemia [19] to 79 % for motility studies [20–29].

Such variation in opinion and perceived indications may depend on the wide experimental potential for MRE as well as the academic orientation of our experts. These experts are more known for their research and inclined to test new applications. Experience may also contribute to variation in the responses we observed [30, 31]. Small bowel diseases are varied, many are relatively uncommon, and it is time-consuming for each research group to accrue sufficient numbers of cases and report them, and for other researchers to confirm the initial observations. Only Crohn’s disease has been studied in large numbers and by many individuals and groups. Oftentimes CT of the small intestine is preferred to MRE because of the availability and lower cost. MRE, however, offers diagnostic advantages in addition to a lack of ionising radiation, and MRE can also be performed without intravenous contrast agents if there is renal insufficiency [32].

We did not focus on the technique and imaging sequences required for MRE, which deserves a separate study. The amount of enteral agent [33], its timing [34], osmolarity [35], type of agent [36, 37], its concentration [38], type of antiperistaltic agent [39, 40], additives such as erythromycin and enema [41], MRI sequences used [42, 43] and field strength [44] are just some possible modifications. Unfortunately most if not all protocol modifications have been undertaken on healthy volunteers. Some areas have not been touched. One area specifically not covered was administration of intravenous contrast agents as this was not expected to change because of GA. The authors had been asked to mention what parts of the protocol they would change if performing MRE under GA. Colon distention, though shown to improve small bowel distention, is rarely used. Furthermore, employing an enema in an unconscious patient inside an MR machine is probably never used. Nonetheless no one reported enema administration when asked about protocol modifications due to patients undergoing GA. Among the questions we posed, there was no single factor that was considered by at least 80 % of our experts as important for determining the volume and/or rate of the agent administered. The factors that the experts agreed upon were that the signal characteristic of the agent was biphasic, behaving like water, and that the patient was examined prone.

We, however, focused more on the choice between MREc and MREg as this has been more controversial and more studied, albeit only in patients with Crohn’s disease [45, 46]. In brief patients prefer MREg [46], while radiologists prefer MREc images [46–50]. MREc probably demonstrates more superficial ulcerations than MREg [18]. The joint position statement from ECCO and ESGAR also considered enteroclysis as likely better for the diagnosis of partial small bowel obstruction in Crohn’s disease [2], though no statistically significant advantage was evident in the studies referenced [50]. For other patients, such as those with malignancies or gastrointestinal bleeding, every tumour site is important. This is probably reflected in higher proportions of our experts choosing MREc than any other indication, though still less than MREg. Studies such as that from Gupta et al. [51] with MREg have shown such good results that one can understand why experts prefer MREg. In this particular study, recruiting patients with Peutz-Jeghers syndrome, patients preferred capsule endoscopy over MREg and one might speculate that this preference would be more exaggerated if MREc had been offered.

Half of our experts had been exposed to the scenario of MRE under GA, an observation regardless of whether MRI under GA was available at their own centre. Most experts when confronted with this dilemma still opted for MRI, but used a standard approach. This choice suggests that MRI is such a powerful method that most experts still consider it advantageous to do MRI as opposed to choosing another modality.

The change in methods due to GA can be based on two aspects. One is that MRI may not be much better than other available methods (e.g. CT) that could easily replace MRI. The other is that SB distention is not important and the risk of SB distention outweighs any potential benefits. In some cases such as unknown GI bleeding no one would risk MRI with, or without, SB distention as the first choice. For diagnosis and follow-up of SB Crohn’s disease and motility disturbances, however, there are some who consider that the benefits of MRI outweigh its risk. Our study demonstrates that the widespread use of MRE has led to requests for it in patients who otherwise require GA under anaesthesia. Considering the risks associated with GA, therefore, it seems prudent to employ other available modalities such as CT if the benefit from MRI does not justify the risk of GA. If the benefit of MRI seems to outweigh the risk of GA, then a decision must be made on how valuable the distention is. Finally, if distention is required and MRE is employed, then based on the experience of radiologists in this study, MREc is the preferred method, avoiding major filling of the stomach and having more control over gastric filling (perhaps via a separate gastric tube).

Our study has several shortcomings. First, the selection process of the experts could be questioned, i.e. who qualifies as an expert. Most of our researchers are from the Western hemisphere and Australia. Clearly they have more experience with Crohn’s disease than certain other diseases, e.g. small bowel tuberculosis. Despite this, the variation in responses was so striking that we expect that with the inclusion of more international experts with more heterogeneous backgrounds this variation would only become even more prominent. Second, it has become common practice to use the Delphi process, or similar approaches, for examining agreement among experts. Our main concern was centred on MRE under GA and other areas were touched on only briefly. For many potential indications there are no existing publications in the available literature and so the Delphi process (or its modifications) and/or systematic review and meta-analysis are impossible.

## Conclusion

Most experts clearly prefer MREg over MREc. There are many emerging indications for MRE. When confronted by the prospects of performing MRE under GA many experts would still do MRI with or without small bowel distention rather than choosing another modality. This article, however, demonstrates that more work is needed to address the fundamental issues concerning MRE. MRE is usually performed by a few enthusiastic individuals working at separate centres and there is a need for more collaborative work putting the ideas and experiences of these individuals together.

## Abbreviations

CT: Computerised tomography
ECCO: European Crohn’s and Colitis Organisation
EL: Evidence level
ESGAR: European Society of Gastrointestinal and Abdominal Radiology
GA: General anaesthesia
MRE: Magnetic resonance imaging of small bowel
MREc: Magnetic resonance imaging performed by means of enteroclysis
MREg: Magnetic resonance imaging performed by means of oral intake (or nasogastric tube), also called enterography follow-through or hydro-MRI
MRI: Magnetic resonance imaging
NG: Nasogastric
SB: Small bowel

## References

[1] Panes J, Bouhnik Y, Reinisch W, Stoker J, Taylor SA, Baumgart DC (2013). Imaging techniques for assessment of inflammatory bowel disease: joint ECCO and ESGAR evidence-based consensus guidelines. J Crohn’s Colitis.

[2] Arlachov Y, Ganatra RH (2012). Sedation/anaesthesia in paediatric radiology. Br J Radiol.

[3] Shankar VR (2008). Sedating children for radiological procedures: an intensivist’s perspective. Pediatr Radiol.

[4] Dalal PG, Murray D, Cox T, McAllister J, Snider R (2006). Sedation and anesthesia protocols used for magnetic resonance imaging studies in infants: provider and pharmacologic considerations. Anesth Analg.

[5] Schulte-Uentrop L, Goepfert MS (2010). Anaesthesia or sedation for MRI in children. Curr Opin Anaesthesiol.

[6] Hafeez R, Punwani S, Boulos P, Bloom S, McCartney S, Halligan S (2011). Diagnostic and therapeutic impact of MR enterography in Crohn’s disease. Clin Radiol.

[7] Steward MJ, Punwani S, Proctor I, Adjei-Gyamfi Y, Chatterjee F, Bloom S (2012). Non-perforating small bowel Crohn’s disease assessed by MRI enterography: derivation and histopathological validation of an MR-based activity index. Eur J Radiol.

[8] Hafeez R, Greenhalgh R, Rajan J, Bloom S, McCartney S, Halligan S (2011). Use of small bowel imaging for the diagnosis and staging of Crohn’s disease—a survey of current UK practice. Br J Radiol.

[9] Tolan DJ, Greenhalgh R, Zealley IA, Halligan S, Taylor SA (2010). MR enterographic manifestations of small bowel Crohn disease. Radiographics.

[10] Punwani S, Rodriguez-Justo M, Bainbridge A, Greenhalgh R, De Vita E, Bloom S (2009). Mural inflammation in Crohn disease: location-matched histologic validation of MR imaging features. Radiology.

[11] Casciani E, Masselli G, Di Nardo G, Polettini E, Bertini L, Oliva S (2011). MR enterography versus capsule endoscopy in paediatric patients with suspected Crohn’s disease. Eur Radiol.

[12] Masselli G, Casciani E, Polettini E, Lanciotti S, Bertini L, Gualdi G (2006). Assessment of Crohn’s disease in the small bowel: prospective comparison of magnetic resonance enteroclysis with conventional enteroclysis. Eur Radiol.

[13] Masselli G, Brizi MG, Menchini L, Minordi L, Vecchioli SA (2005). Magnetic Resonance Enteroclysis imaging of Crohn’s. Radiol Med.

[14] Frøkjaer JB, Larsen E, Steffensen E, Nielsen AH, Drewes AM (2005). Magnetic resonance imaging of the small bowel in Crohn’s disease. Scand J Gastroenterol.

[15] Lawrance IC, Welman CJ, Shipman P, Murray K (2009). Correlation of MRI-determined small bowel Crohn’s disease categories with medical response and surgical pathology. World J Gastroenterol.

[16] Ziech ML, Bossuyt PM, Laghi A, Lauenstein TC, Taylor SA, Stoker J (2012). Grading luminal Crohn’s disease: which MRI features are considered as important?. Eur J Radiol.

[17] Bickelhaupt S, Froehlich JM, Cattin R, Patuto N, Tutuian R, Wentz KU (2013). Differentiation between active and chronic Crohn’s disease using MRI small-bowel motility examinations—initial experience. Clin Radiol.

[18] Torkzad MR, Ullberg U, Nyström N, Blomqvist L, Hellström P, Fagerberg UL (2012). Manifestations of small bowel disease in pediatric Crohn’s disease on magnetic resonance enterography. Inflamm Bowel Dis.

[19] Lauenstein TC, Ajaj W, Narin B, Göhde SC, Kröger K, Debatin JF (2005). MR imaging of apparent small-bowel perfusion for diagnosing mesenteric ischemia: feasibility study. Radiology.

[20] Froehlich JM, Patak MA, von Weymarn C, Juli CF, Zollikofer CL, Wentz KU (2005). Small bowel motility assessment with magnetic resonance imaging. J Magn Reson Imaging.

[21] Bickelhaupt S, Pazahr S, Chuck N, Blume I, Froehlich JM, Cattin R (2013). Crohn’s disease: small bowel motility impairment correlates with inflammatory-related markers C-reactive protein and calprotectin. Neurogastroenterol Motil.

[22] Bickelhaupt S, Froehlich JM, Cattin R, Raible S, Bouquet H, Bill U (2014). Software-assisted small bowel motility analysis using free-breathing MRI: feasibility study. J Magn Reson Imaging.

[23] Cullmann JL, Bickelhaupt S, Froehlich JM, Szucs-Farkas Z, Tutuian R, Patuto N (2013). MR imaging in Crohn’s disease: correlation of MR motility measurement with histopathology in the terminal ileum. Neurogastroenterol Motil.

[24] Menys A, Atkinson D, Odille F, Ahmed A, Novelli M, Rodriguez-Justo M (2012). Quantified terminal ileal motility during MR enterography as a potential biomarker of Crohn’s disease activity: a preliminary study. Eur Radiol.

[25] Menys A, Taylor SA, Emmanuel A, Ahmed A, Plumb AA, Odille F (2013). Global small bowel motility: assessment with dynamic MR imaging. Radiology.

[26] Menys A, Helbren E, Makanyanga J, Emmanuel A, Forbes A, Windsor A (2013). Small bowel strictures in Crohn’s disease: a quantitative investigation of intestinal motility using MR enterography. Neurogastroenterol Motil.

[27] Torkzad MR, Vargas R, Tanaka C, Blomqvist L (2007). Value of cine MRI for better visualization of the proximal small bowel in normal individuals. Eur Radiol.

[28] Yacoub JH, Oto A (2014). New magnetic resonance imaging modalities for Crohn disease. Magn Reson Imaging Clin N Am.

[29] Yacoub JH, Obara P, Oto A (2013). Evolving role of MRI in Crohn’s disease. J Magn Reson Imaging.

[30] Negaard A, Mulahasanovic A, Reisaeter LA, Aasekjaer K, Sandvik L, Klow NE (2008). Crohn’s disease evaluated with magnetic resonance enteroclysis: diagnostic performance of experienced and inexperienced readers before and after training. Acta Radiol.

[31] Negaard A, Sandvik L, Mulahasanovic A, Berstad AE, Klöw NE (2006). Magnetic resonance enteroclysis in the diagnosis of small-intestinal Crohn’s disease: diagnostic accuracy and inter- and intra-observer agreement. Acta Radiol.

[32] Masselli G, Gualdi G (2013). CT and MR enterography in evaluating small bowel diseases: when to use which modality?. Abdom Imaging.

[33] Kinner S, Kuehle CA, Herbig S, Haag S, Ladd SC, Barkhausen J (2008). MRI of the small bowel: can sufficient bowel distension be achieved with small volumes of oral contrast?. Eur Radiol.

[34] Kuehle CA, Ajaj W, Ladd SC, Massing S, Barkhausen J, Lauenstein TC (2006). Hydro-MRI of the small bowel: effect of contrast volume, timing of contrast administration, and data acquisition on bowel distention. AJR Am J Roentgenol.

[35] Ajaj W, Goyen M, Schneemann H, Kuehle C, Nuefer M, Ruehm SG (2005). Oral contrast agents for small bowel distension in MRI: influence of the osmolarity for small bowel distention. Eur Radiol.

[36] Ajaj W, Goehde SC, Schneemann H, Ruehm SG, Debatin JF, Lauenstein TC (2004). Oral contrast agents for small bowel MRI: comparison of different additives to optimize bowel distension. Eur Radiol.

[37] Ajaj W, Goehde SC, Schneemann H, Ruehm SG, Debatin JF, Lauenstein TC (2004). Dose optimization of mannitol solution for small bowel distension in MRI. J Magn Reson Imaging.

[38] Lauenstein TC, Schneemann H, Vogt FM, Herborn CU, Ruhm SG, Debatin JF (2003). Optimization of oral contrast agents for MR imaging of the small bowel. Radiology.

[39] Gutzeit A, Binkert CA, Koh DM, Hergan K, von Weymarn C, Graf N (2012). Evaluation of the anti-peristaltic effect of glucagon and hyoscine on the small bowel: comparison of intravenous and intramuscular drug administration. Eur Radiol.

[40] Froehlich JM, Daenzer M, von Weymarn C, Erturk SM, Zollikofer CL, Patak MA (2009). Aperistaltic effect of hyoscine N-butylbromide versus glucagon on the small bowel assessed by magnetic resonance imaging. Eur Radiol.

[41] Lauenstein TC, Vogt FM, Herborn CU, DeGreiff A, Debatin JF, Holtmann G (2003). Time-resolved three-dimensional MR imaging of gastric emptying modified by IV administration of erythromycin. AJR Am J Roentgenol.

[42] Neubauer H, Pabst T, Dick A, Machann W, Evangelista L, Wirth C (2013). Small-bowel MRI in children and young adults with Crohn disease: retrospective head-to-head comparison of contrast-enhanced and diffusion-weighted MRI. Pediatr Radiol.

[43] Maccioni F, Patak MA, Signore A, Laghi A (2012). New frontiers of MRI in Crohn’s disease: motility imaging, diffusion-weighted imaging, perfusion MRI, MR spectroscopy, molecular imaging, and hybrid imaging (PET/MRI). Abdom Imaging.

[44] Patak MA, von Weymarn C, Froehlich JM (2007). Small bowel MR imaging: 1.5T versus 3T. Magn Reson Imaging Clin N Am.

[45] Torkzad MR, Lauenstein TC (2009). Enterclysis versus enterography: the unsettled issue. Eur Radiol.

[46] Lawrance IC, Welman CJ, Shipman P, Murray K (2009). Small bowel MRI enteroclysis or follow through: which is optimal?. World J Gastroenterol.

[47] Negaard A, Sandvik L, Berstad AE, Paulsen V, Lygren I, Borthne A (2008). MRI of the small bowel with oral contrast or nasojejunal intubation in Crohn’s disease: randomized comparison of patient acceptance. Scand J Gastroenterol.

[48] Masselli G, Casciani E, Polettini E, Gualdi G (2008). Comparison of MR enteroclysis with MR enterography and conventional enteroclysis in patients with Crohn’s disease. Eur Radiol.

[49] Masselli G, Vecchioli A, Gualdi GF (2006). Crohn disease of the small bowel:MR enteroclysis versus conventional enteroclysis. Abdom Imaging.

[50] Negaard A, Paulsen V, Sandvik L, Berstad AE, Borthne A, Try K (2007). A prospective randomized comparison between two MRI studies of the small bowel in Crohn’s disease, the oral contrast method and MR enteroclysis. Eur Radiol.

[51] Gupta A, Postgate AJ, Burling D, Ilangovan R, Marshall M, Phillips RK (2010). A prospective study of MR enterography versus capsule endoscopy for the surveillance of adult patients with Peutz-Jeghers syndrome. AJR Am J Roentgenol.

